# Impact of complementary and alternative medicines on antiepileptic medication adherence among epilepsy patients

**DOI:** 10.1186/s12906-021-03224-2

**Published:** 2021-02-04

**Authors:** Muhammad Junaid Farrukh, Mohd Makmor-Bakry, Ernieda Hatah, Tan Hui Jan

**Affiliations:** 1 Faculty of Pharmacy, Universiti Kebangsaan Malaysia, Jalan Raja Muda Abdul Aziz, 50300 Kuala Lumpur, Malaysia; 2 Faculty of Pharmaceutical Sciences, UCSI University, Kuala Lumpur, Malaysia; 3 Faculty of MedicinePusat Perubatan Universiti Kebangsaan Malaysia (PPUKM), Kuala Lumpur, Malaysia

**Keywords:** Medication adherence, AED, Epilepsy, Complementary and alternative medicine, Supplements

## Abstract

**Background:**

The aim of this study was to assess the knowledge, attitude, and practice of complementary and alternative medicine (CAM) and its impact on antiepileptic drug (AED) adherence among patients with epilepsy.

**Methods:**

A cross-sectional study was carried out on 100 epilepsy patients, aged 18 years or older that did not have any physical or psychiatric illness. A patient-administered questionnaire was used to assess their knowledge, attitude towards, practice, and perceived effectiveness (KAPP) of CAM. Established adherence assessment tools were used to determine patient medication adherence.

**Results:**

The prevalence of CAM usage was found to be at 58%. CAM was used more frequently by males (*n* = 32, 60.4%) than by females (*n* = 26, 55.3%; *p* = 0.609). The most commonly used CAM included vitamins and minerals (36%), ginseng (16%), antioxidants (15%), and acupuncture (12%). A significant number of patients had low knowledge of (59%) and a positive attitude (54%) toward complementary and alternative medicine. Main reasons for using CAM were a lower price, better availability, and inadequate seizure control by AEDs. About 43% of the patients who used CAM informed their doctor. Prevalence of non-adherence to AED therapy was found to be 68%. A significant association was found between non-adherence and CAM usage (*p* < 0.01).

**Conclusion:**

A high prevalence of CAM usage and non-adherence to AEDs among epilepsy patients was identified. CAM usage was associated with a non-adherence to AED therapy. This study highlights the need to explore CAM usage with patients before making clinical decisions to achieve the best outcomes from AED therapy.

**Supplementary Information:**

The online version contains supplementary material available at 10.1186/s12906-021-03224-2.

## Background

Epilepsy is a chronic disease of the brain that can affect individuals at any age. Nearly 50 million people worldwide have epilepsy, making it one of the most common neurological disorders [1]. Most seizures can be controlled by medications called antiepileptic drugs (AEDs) [2]. Complementary and alternative medicine (CAM) is used by epileptic patients as an alternative treatment option however the effectiveness of some of them has not been established using scientific methods [3]. Globally, the percentage of CAM usage among patients with epilepsy was reported between 7.3 and 73.3% [3]. The most common CAM remedies used in the treatment of epilepsy include mind-body therapies, such as reiki and yoga; biologic-based therapies, such as herbal remedies, dietary supplements, and homeopathy; and manipulative-based therapies, such as chiropractic [4]. The type of CAM used may vary due to differences in cultural norms and healthcare settings.

In Malaysia, CAM use is prevalent among 71.2% of the general public [5]. There are several issues with CAM use. The Malaysia Drug Control Authority (DCA) is responsible for protecting public health in ensuring the safety and efficacy of pharmaceutical and biological products. However, the safety and effectiveness of CAM are not fully regulated by the DCA [6]. CAM may cause a drug-drug interaction which may increase the risk of seizures through several mechanisms, such as intrinsic proconvulsant properties, contamination by heavy metals, effects on the cytochrome P450 enzymes and P-glycoproteins and altering antiepileptic drug (AED) disposition. Such interactions may be difficult to predict, since the quality and quantity of active ingredients are often unknown. According to a study, 50% of the patients using a CAM-based medicine had an increase in their seizures [7]. Most patients are reluctant to inform their physicians about taking a CAM based medicine as they do not consider such alternative medicines as drugs [8].

In a systematic review, the worldwide AED non-adherence rate among patients with epilepsy was reported to be between 25 and 66% [3]. In Malaysia, the prevalence of poor adherence to AEDs has been reported as 64.1% of all epileptic patients [9]. Non-adherence to medication is a serious issue in patients with epilepsy. The belief that epilepsy has a spiritual or psychological cause contributes to inadequate AED therapy and a higher dependence on CAM [10]. Although several studies have reported the reasons for CAM usage among epilepsy patients, the global data on evidence of AED non-adherence and its association with CAM usage is scarce. Therefore, the objective of this study was to determine the prevalence of CAM usage, to evaluate the knowledge, attitude towards, and practice of CAM and its impact on AED adherence among patients with epilepsy.

## Methods

### Study design

We conducted a cross-sectional study on 100 patients diagnosed with epilepsy to evaluate the knowledge, attitude towards, practice, and perceived effectiveness of CAM. The patients were recruited from the neurology clinic at the Hospital University Kebangsaan Malaysia.

### Sample size

Sample size was calculated based on descriptive survey using Raosoft Calculator (Raosoft Inc. 2004). The margin of error and confidence interval were set at 5 and 95%, respectively. Response distribution was set at 50% to get the higher sample size. Population size was estimated at 150 for epilepsy patient within the duration of study as the clinic was run 2 days a week. Thus, a total of 109 patients was estimated as the sample size.

### Study setting

The patients were recruited from the neurology clinic at the Hospital Universiti Kebangsaan Malaysia from March 2017 to November 2018.

### Sampling method

Data was collected using simple random sampling. First the appointment list of patients was obtained from the clinic appointment record and then patients were randomly selected using a random number generator. Random Number Generator®, Android App developed by Ux Apps will be used (Random Number Generator 2016). Patients were recruited based on the following criteria.

### Inclusion criteria

Epilepsy patients who were 18 years or older and had been on an AED for at least 6 months, without any documented physical or psychiatric illness such as schizophrenia and major depression were included.

### Assessment tools

The questionnaires comprised of seven main sections which were adapted from previous studies [11, 12]. Section A captured basic socio-demographic data, including age, sex, religion, marital status, level of education, monthly income, number of comorbidities, and number of medications. Section B consisted of knowledge about CAM (4 questions), with a correct answer scored as 1 (maximum score = 4). Section C covered the types of CAM and their perceived effectiveness of CAM. Section D and Section E consisted of attitude (8 questions) and the practice of CAM (8 questions), respectively. It was modified and translated into the Malay language using the forward-backward translation method. Content validity was assessed by five experts (physician, academics, and pharmacists). A pilot study was first carried out on 30 epileptic patients to ensure the reliability of the questionnaire formulated. The internal consistency was calculated using Cronbach’s alpha coefficient, which was 0.7 for knowledge, 0.73 for attitude, and 0.85 for practice. Section F consisted of the Malaysian Medication Adherence Scale (MALMAS) [13] and the Medication Possession Ratio (MPR) [14] so as to assess adherence. The MPR was determined from our pharmacy information system (PIS) by calculating the number of days’ supply of medication dispensed divided by the number of days between the first and last prescription refill. If any of the two tools showed non-adherence, patients were considered as non-adherent.

### Ethical considerations

Ethical approval was obtained from the UKMC ethical committee, reference no. UKM PPI/111/8/JEP-2017-138. Written informed consent was obtained from all patients before participation in the study.

### Data collection

**Fig. 1:**
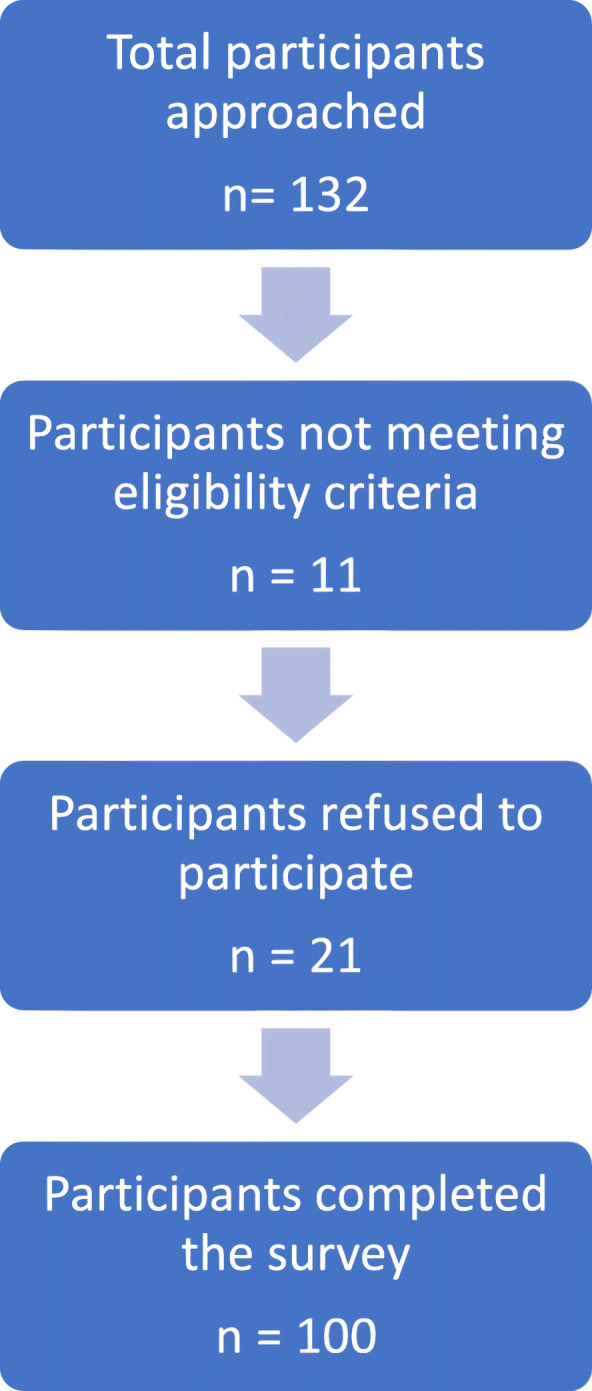
CONSORT flow diagram. CONSORT flow diagram showing participants recruitment process. a total of 132 patients were approached and out of them 21 patients refused to participate. The response rate of the patients was 84.09%

The patients were identified for participation in the study by a physician. Patients were informed about the purpose of this research. When appropriate, nurses also assisted patients filling in the questionnaires. CONSORT flow diagram showing participants recruitment process is presented as Fig. 1.

### Statistical analyses

Statistical analyses were performed using the IBM SPSS Statistics Version 23. To analyze categorical variables frequencies and percentages were used. For continuous variables describing the study population, descriptive statistics, including the mean and standard deviation (SD), were used. For the knowledge questions asked, each question had two possible responses (‘yes’ and ‘no’). A scoring method was used for analysis. A correct answer was given a score of 1, while an incorrect answer was scored 0. For the attitude and practice questions, a Likert rating scale of five points was used. There were seven attitude questions, each question was given a score as follows: Strongly agree = 5, Agree = 4, Not sure = 3, Disagree = 2 and Strongly disagree = 1. Question 8 was given a reverse score. For the six practice questions, each question was given a score as follows: Strongly agree = 1, Agree = 2, Not sure = 3, Disagree = 4 and Strongly disagree = 5. Questions 3 and 5 were given a reverse score. The mean of the total scores for knowledge and practice was used to determine the midpoint of ‘good knowledge’, ‘positive attitude’ and ‘good practice’ [15]. Univariate analysis to study the relationship between the variables and CAM usage was done using Chi-square test and Independent t-test where necessary. Association between adherence and CAM usage was analysed using Chi-square test. Variables with *p* < 0.05 was considered as statistically significant *p* value.

## Results

For this survey, a total of 132 patients were approached and out of them 21 patients refused to participate. The response rate of the patients was 84.09%. The reasons given by the patients for not participating in this survey were time constraints (*n* = 8) and unwilling to participate (*n* = 13). In total, 47 females and 53 males between the ages of 18 and 79 years old participated in our study. The mean age was 40.18 (SD 17.9) years old. The prevalence of CAM use was found to be 58% among the studied population. CAM use was more frequent in males (*n* = 32, 60.4%)as compared to females (*n* = 26, 55.3%) however, this difference was not significant. The majority of patients were Malay (*n* = 51), and Chinese (*n* = 36), while the remaining patients were Indian (*n* = 13). Patients represented varying education levels, ranging from having had no formal education to a post-graduate qualification. The number of comorbidities varied among the patients (range between 0 to 4) with mean of 1.87 (SD 1.01). Seventy-nine percent of patients reported that they had heard of CAM. The primary source of information on CAM were friends (39%), family (27%), internet (19%) and doctor (15%). The socio-demographic characteristics of the patients and their profile of CAM use are presented in Table 1.

**Table 1:** Socio-demographic characteristics and CAM Usage

Item	Overall	CAM User	CAM Non-User	Statistics
Age in years, mean (SD)	40.18 (17.9)	47.19 (17.6)	43.05 (16.0)	0.232^a^
Sex, n (%)				
Male	53 (53)	32 (60.4)	21 (39.6)	0.609^b^
Female	47 (47)	26 (55.3)	21 (44.7)	
Race, n (%)				
Malay	51 (51)	26 (51)	25 (49)	0.214^b^
Chinese	36 (36)	22 (61.1)	14 (38.9)	
Indian	13 (13)	10 (76.9)	3 (23.1)	
Education Level, n (%)				
No formal education	2 (2)	0 (0)	2 (100)	0.08^b^
Primary school	18 (18)	12 (66.7)	6 (33.3)	
Secondary school	48 (48)	30 (62.5)	18 (37.5)	
Diploma	6 (6)	4 (66.7)	2 (33.3)	
Undergraduate	20 (20)	7 (35)	13 (65)	
Post-graduate	6 (6)	5 (83.3)	1 (16.7)	
Marital Status, n (%)				
Single	32 (61.5)	20 (38.5)		0.751^b^
Married	25 (54.3)	21 (45.7)		
Divorced	1 (50)	1 (50)		
Number of co-morbidities, mean (SD)	1.87 (1.01)	2.0 (1.0)	1.69 (0.9)	0.132^a^

a = Independent t-test; b = Chi-square test

The most commonly used CAM included vitamins and minerals (*n* = 36, 62%), ginseng (*n* = 16, 27.5%), antioxidants (*n* = 15, 25.8%) and acupuncture (*n* = 12, 20.6%). The majority (68.1%) of patients reported that CAM was effective. The main reasons for using CAM were that it was cheaper, better availability and due to inadequate seizure control by AEDs. Only 43.1% of the patients who were using CAM revealed the practice to their doctors. A significant number of patients had low knowledge and a positive attitude towards complementary and alternative medicine. The distribution and perceived effectiveness of CAM usage, as reported by the patients, is summarized in Table 2.

**Table 2:** Distribution and perceived effectiveness of CAM usage

Types of CAM used	% of user, n (%)	% of those perceived effective, n (%)
**Mind and Body practice (*n*** **= 47)**		
Acupuncture	12 (20.6)	6 (50)
Aromatherapy	5 (8.6)	4 (80)
Cupping	5 (8.6)	3 (60)
Massage	12 (20.6)	6 (50)
Prayer for health	5 (8.6)	4 (80)
Structured Exercise	8 (13.7)	2 (25)
**Natural Products (*n*** **= 79)**		
Antioxidants	15 (25.8)	11 (73.3)
Detoxifying diet	2 (3.4)	1 (50)
Ginseng	16 (27.5)	12 (75)
Spirulina	4 (6.8)	4 (100)
Unknown Herbal	6 (10.3)	4 (100)
Vitamins and minerals	36 (62)	23 (63.8)
Others		
**Traditional Medicine (*n*** **= 22)**		
Homeopathy	3 (5.17)	3 (100)
Traditional Chinese Medicine	8 (13.7)	8 (100)
Traditional Indian Medicine	4 (6.89)	2 (50)
Traditional Malay Medicine	7 (12)	5 (71.4)

The average knowledge, attitude, and practice scores were determined as 2.09 (SD 1.3), 28.3 (SD 5.4) and 21.3 (SD 8.0), respectively. The majority of patients showed poor knowledge (59%), positive attitude (54%), and poor practices of CAM (51.7%). A significant difference was identified in the mean attitude score between CAM users and non-users (*p* = 0.05). The attitude score showed a negative correlation with CAM practice (*r* = − 0.404, *p* < 0.01). Patients with a higher score in knowledge displayed better CAM practices (*r* = 0.347, *p* = 0.05). This data is summarized in Table 3.

**Table 3:** Predictivity of attitude statements on CAM usage

Characteristics	CAM User	CAM Non-User	***p***-value	OR	95% CI
CAM provides quick and additional relief					
Yes	53 (67.1)	26 (32.9)	< 0.001	6.523	2.15–19.17
No	5 (23.8)	16 (76.2)			
CAM is cheaper compared to modern medicine					
Yes	54 (62.1)	33 (37.9)	0.03	3.6	1.05–12.09
No	4 (30.8)	9 (69.2)			
CAM provides a permanent cure					
Yes	50 (72.5)	19 (27.5)	< 0.001	7.5	2.8–19
No	8 (25.8)	23 (74.2)			
People use CAM because it is easily available					
Yes	50 (72.5)	19 (27.5)	< 0.001	7.5	2.8–19
No	8 (25.8)	23 (74.2)			
CAM providers give good information about CAM					
Yes	48 (67.6)	23 (32.4)	0.002	3.9	1.5–9.8
No	10 (34.5)	19 (65.5)			
CAM promotes self-healing					
Yes	51 (69.9)	22 (30.1)	< 0.001	6.62	2.4–17.9
No	7 (25.9)	20 (74.1)			
People use CAM due to fearing discomfort from allopathic medicines					
Yes	42 (76.4)	13 (23.6)	< 0.001	5.8	2.4–13.9
No	16 (35.6)	29 (64.4)			
CAM should be used only in minor illness					
Yes	12 (33.3)	24 (66.7)	< 0.001	0.196	0.08–0.47
No	46 (71.9)	18 (28.1)			

### Adherence to AED

The prevalence of AED non-adherence was 68%. There was no significant difference between adherence status and most of the sociodemographic indicators. However, it was found that patients who were married (*p* = 0.052) and had a higher level of education showed good adherence (*p* < 0.01). The mean difference in the number of comorbidities and the number of medicines was higher among non-adherent patients (*p* < 0.01).

Reasons of non-adherence reported by the patients were forgetfulness (*n* = 38), missing a dose when away from home (*n* = 46), difficulty in taking medicine (*n* = 45), problem in remembering to take medicine (*n* = 33) and stopping medicines after feeling better (*n* = 15). Patients who were adherent to AED showed good knowledge, poor attitude, and good practice towards CAM usage. Comparison of knowledge, attitude, and practice of CAM scores and adherence status are summarized in Table 4.

**Table 4:** Comparison of Knowledge, Attitude, and Practice of CAM scores and adherence status

Item (Range of score)	Descriptive, Mean (SD)	Adherent	Non-Adherence	***p***-values
**Knowledge (0–4)**	2.09 (1.3)	2.8 (1.2)	1.73 (1.2)	< 0.01^a^
**Attitude (5–40)**	28.3 (5.4)	23.7 (4.7)	30.4 (4.4)	< 0.01^a^
**Practice (5–40)**	21.3 (8.0)	32 (4.2)	19.6 (7.1)	< 0.01^a^

a = Independent t-test

There was a significant relationship seen between medication non-adherence and CAM use (*p* < 0.01). This was seen to be more prevalent among patients with a lower education level. The relationship between CAM use and medication adherence is shown in Table 5.

**Table 5:** Relationship between CAM usage and medication adherence

Item	Adherent	Non-Adherent	***p***-value
**CAM user**	8	50	< 0.01^a^
**CAM Non-user**	24	18	

a = Chi-square test

## Discussion

Epilepsy is a stigmatizing neurological disorder that often results in significant physical, psychological, and financial burden on both individuals and families [16]. Problems associated with epilepsy are further aggravated when patients neglect to take their antiepileptic drug (AED) therapy and begin depending on other treatment options [17]. The goal of this study was to assess the knowledge, attitude, and practice of complementary and alternative medicine (CAM) and its impact on antiepileptic drug (AED) adherence among patients with epilepsy.

The findings revealed a high prevalence of CAM usage among patients with epilepsy. The prevalence of CAM use among epilepsy patients reported in this study is higher than an earlier study reported in Malaysia [18]. This could be due to having a larger sample size and the comprehensive, detailed categories of CAM used by our study. The global prevalence of CAM use reported in previous studies was between 7.5 and 73.3%. A systematic review on the CAM usage among epilepsy patients reported that CAM is more widely used in developed countries as compared to developing countries. However, the definition of CAM and types of CAM may also vary among developed and developing countries [3]. Although Malaysia is a developing country, the higher prevalence of CAM usage may be due to the multi-racial culture in Malaysia where the people have various treatment choices ranging from culturally specific traditional medicines to modern CAM [3]. Malaysia’s healthcare system is divided into two highly developed sectors, a government-led and funded public sector, and a booming private. The public sector caters to about 65% of the population [19]. Furthermore, the cost of epilepsy medications is very high especially the newer agents as they are imported from other countries and are not easily accessible. Patients with low socioeconomic background might not be able to afford such high cost of new medicine and need to continue with the generic medicine available in the government or public health clinics. A study done on the cost-effectiveness of AEDs for the management of epilepsy in Malaysia reported that they newer agents were considered non-cost-effective in controlling seizures [20]. In Malaysia most of the insurance policies do not cover epilepsy, so it is very difficult for an epilepsy patient who needs AEDs to claim from insurance, so they tend to rely on CAM [21].

The most popular CAM reported in this study were vitamins and minerals, ginseng, antioxidants, and acupuncture. The type of CAM usage varied between different age-groups. Acupuncture, traditional Chinese and Malay medicines were mostly used by elderly patients. The younger population was seen to be widely using vitamins and supplements due to marketing and promotional strategies by companies to attract them. The higher use of CAM in the elderly can be justified by an increased number of chronic diseases, most of which can only be controlled and not cured with current conventional treatment options [22]. The types of CAM used in this study were comparable to previous studies, other than some variation that was caused by differences in cultural and traditional norms. For example, in Western countries, the use of vitamins, herbs, and yoga was more prevalent [23–25]. Prayer for health and amulets was common in Middle-eastern countries [26]. In India, Ayurveda was more frequently practiced [27–29] whereas, in Taiwan, traditional Chinese medicines were seen to be used more frequently [30].

The majority of patients showed poor knowledge and a positive attitude towards CAM usage. The majority of CAM users showed poor practices of CAM. Malay patients and patients with a higher level of education had good knowledge of CAM. Attitude scores showed a negative correlation with the knowledge score, level of education and CAM practice. Patients that displayed high knowledge showed good CAM practices. Similar results were reported in a study carried out in Malaysia among the general population [31]. People who were using CAM showed a stronger belief in it, with a more positive attitude towards CAM than those who were not using it. This may indicate that education is needed to explain both the pros and cons of using CAM. Moreover, if patients are well-educated, they will have a better understanding of the etiology of epilepsy, the importance of AED’s and the pros and cons of using CAM.

Primary reasons for using CAM included a lower price, better availability, and patients not receiving adequate seizure control using AEDs. Most CAM users had a lower level of education. This could explain their poor knowledge but positive attitude scores towards CAM. Based on these results it seems that patients can get CAM easily from pharmacies and retail stores, even though it is not clear how effective CAM products are on specific illnesses such as epilepsy. Pharmacists themselves felt they needed to attain more knowledge about CAM [5]. A study done in Singapore found that 81% of pharmacists thought that they did not possess sufficient skills and knowledge to counsel patients on herbal medicine and 90.5% felt that their education curriculum should include more content on CAM [32].

Another important finding from this study was that CAM users did not disclose the usage of CAM to their doctors. A similar finding has been reported in studies done in Malaysia, Singapore, Australia in which respondents did not reveal the use of CAM and any unwanted side effects from CAM use to their healthcare providers [33–35]. This highlights the need for community pharmacists and doctors to ask patients regarding CAM use and appropriately counsel patients on both CAM use and misuse, as well as possible interactions with AEDs. This may help to improve therapeutic outcomes and minimize interactions with AEDs, therefore improving the patient’s quality of life.

The prevalence of AED non-adherence was found to be 68%. In a systematic review, the global prevalence of non-adherence among epileptic patients ranged between 25 and 66% [3]. Moreover, the overall adherence level in western countries was seen to be higher than in Asian counties. This indicates that people residing in developing countries prefer traditional medicines to modern allopathic medicines due to cultural norms. In a previous study done in Malaysia, poor adherence to AEDs was documented as being 64.1% of patients with epilepsy [9]. Our study had a slightly higher poor adherence rate than this which may be due to the combination of subjective and objective assessment tools utilized compared to the previous study which only used a subjective assessment method. Using a subjective self-reporting method is known to result in over-reporting of good adherence [36].

It was found that patients who were married and had a higher level of education showed good medication adherence. Similar findings were reported in a study where un-married patients were more likely to be non-adherent than married patients [37]. According to a meta-analysis, social support is consistently associated with greater medication adherence [37]. This shows that patients who are married are more adherent towards taking medication as their partners play an important role in reminding them to take their medication and following them up.

The most common reasons for non-adherence were forgetfulness, missing dose when away from home, difficulty in taking medicine, problem in remembering to take medicine, fear of AED side effects and stopping medicines after feeling better. Similar findings were reported in other studies [16, 38–40].

CAM users were to a greater extent, non-adherent as compared to non-CAM users. The majority of CAM users reported concurrent use with allopathic medicines. Comparable results were reported previously in Honduras and Poland where most CAM users were seen to be non-adherent to AEDs [10, 41]. Use of AEDs along with CAM results in complex polypharmacy. This potentially leads to non-adherence as it could contribute to patients forgetting to take all their medicines and skipping doses.

Patients who were non-adherent to AED had poor knowledge, positive attitude and poor practice towards CAM use. This was in-line with the results reported in a study where widespread non-adherence to AEDs was attributed to inadequate education and wide usage of CAM [10]. It is imperative to educate patients about the common etiologies of epilepsy and in most cases that AEDs are an effective treatment.

### Limitations

Although the current study has highlighted a number of significant findings, there are some limitations. We did not record the type of epilepsy and AEDs which the patient was taking and are, therefore, unable to relate this to the potential reasons for AED non-adherence. There were a substantial number of patients who refused to take part in the survey, or were excluded due to critical condition and not fit to be interviewed thus the sample size was relatively small. Further research is needed to generalise these results to different clinical settings and on larger sample size. Moreover, an investigation into the effects of CAM use on epilepsy control would provide insight into the potential benefit and harm of CAM.

## Conclusion

A high prevalence of CAM use and non-adherence to AEDs among epilepsy patients was identified. This high rate of CAM use was seen to impact a patient’s medication adherence. There is a need for patient education related to CAM use and AED therapy. The use of CAM should be explored with patients before clinical decisions are made so as to achieve the best treatment outcomes from AED therapy.

## Supplementary Information

**Additional file 1.** Data collection form.

## Data Availability

The datasets generated during and/or analyzed during the current study are available from the corresponding author on reasonable request.

## Abbreviations

CAM: Complementary and alternative medicine
AED: Antiepileptic drug
KAPP: Knowledge, attitude, practice, and perceived effectiveness
MALMAS: Malaysian medication adherence scale
MPR: Medication possession ratio
