# Supplementary material for: Impact of complementary and alternative medicines on antiepileptic medication adherence among epilepsy patients
Source: BMC Complement Med Ther. 2021 Feb 4;21:50. doi: 10.1186/s12906-021-03224-2 (PMC7863518; doi:10.1186/s12906-021-03224-2)
Supplement: Supplementary file 1 — Additional file 1. Data collection form. [file 12906_2021_3224_MOESM1_ESM.docx]

**DATA COLLECTION FORM**


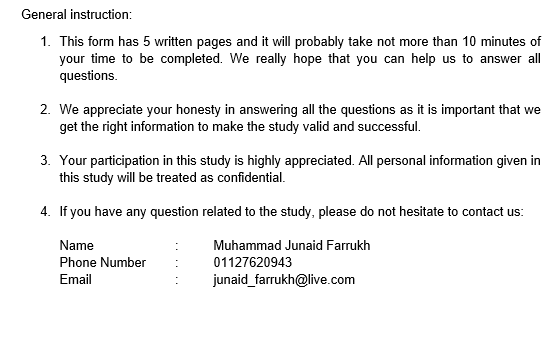

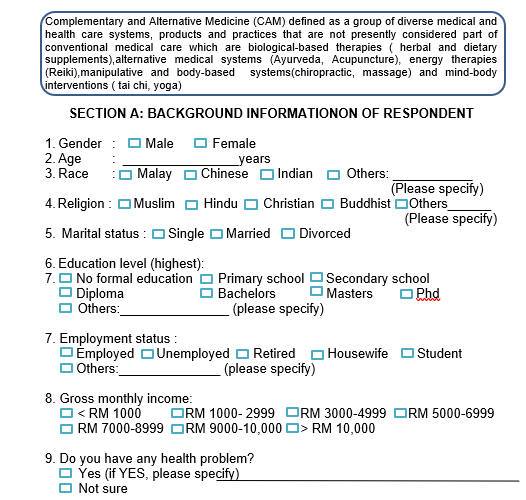


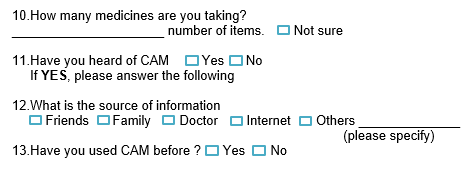


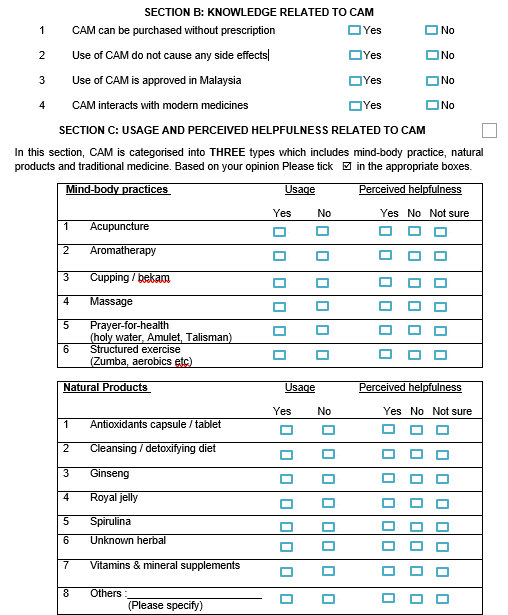


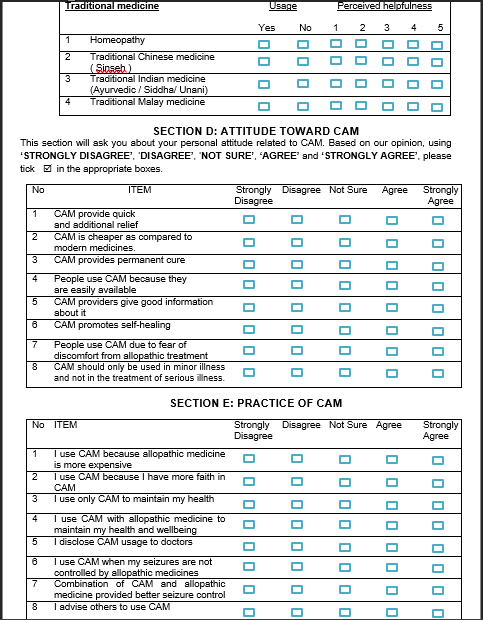


Section: F

| **BMQ- Epilepsy Specific** | | | | | |
| --- | --- | --- | --- | --- | --- |
| 6- item AED- Necessity Scale | | | | | |
|  | Strongly Disagree | Disagree | Uncertain | Agree | Strongly Agree |
| I would be very ill without epilepsy medication |  |  |  |  |  |
| I would prefer to take the epilepsy medication than having the risk of seizure |  |  |  |  |  |
| My life would be difficult without epilepsy medicine |  |  |  |  |  |
| My health in the future is dependent on my epilepsy medication |  |  |  |  |  |
| My life now is dependent on my epilepsy medication |  |  |  |  |  |
| The epilepsy medication helps my health from becoming worse |  |  |  |  |  |
| 10-item AED –Concerns Scale | | | | | |
| I sometimes worry on the long term effect of the epilepsy medication |  |  |  |  |  |
| I sometimes worry that the epilepsy medicine slow me down |  |  |  |  |  |
| I have sufficient information on the epilepsy medication |  |  |  |  |  |
| My epilepsy medicines are a mystery to me |  |  |  |  |  |
| I feel I am labelled ‘sick’ because I take this medicines |  |  |  |  |  |
| I sometimes worry that this medicine impacts my relationship with others |  |  |  |  |  |
| Having to take epilepsy medicine worries me |  |  |  |  |  |
| I sometimes worry that I am too dependent on the medicine |  |  |  |  |  |
| The epilepsy medicine causes unpleasant side effects |  |  |  |  |  |
| The epilepsy medicine disrupts my life |  |  |  |  |  |

Section: G

| Scale | Item | No  problem | Mild problem | Moderate  problem | Serious  Problem |
| --- | --- | --- | --- | --- | --- |
| Fatigue | I am less enthusiastic about day to day activities |  |  |  |  |
| Slowing | My mind does not work as fast as it should |  |  |  |  |
| Memory | I have difficulties remembering names of people |  |  |  |  |
| Concentration | I have difficulties following a book or a film |  |  |  |  |
| Motor co- | I feel clumsy |  |  |  |  |
| Language | I have problems finding the correct word |  |  |  |  |
| Fatigue | I am less capable of undertaking initiatives |  |  |  |  |
| Slowing | My thinking has slowed down |  |  |  |  |
| Memory | I forget things, for example an appointment or where I put an object |  |  |  |  |
| Concentration | I have difficulties concentrating on the things I am doing |  |  |  |  |
| Motor co- | I cannot use a pen or pencil accurately |  |  |  |  |
| Language | I have problems understanding what I read |  |  |  |  |
| Fatigue | I get tired easily and have little energy |  |  |  |  |
| Slowing | It takes me longer to do day to day things |  |  |  |  |
| Memory | I forget things that people have said to me |  |  |  |  |
| Concentration | I can’t concentrate for more than a short period of time |  |  |  |  |
| Motor co- | I constantly bump against tales, doorposts, etc |  |  |  |  |
| Fatigue | I feel worn out |  |  |  |  |
| Slowing | It costs more time for me to get started |  |  |  |  |
| Memory | I get confused and forget what I was doing |  |  |  |  |
| Concentration | I get distracted more easily |  |  |  |  |
| Language | I sometimes stutter or am unable to find the correct words |  |  |  |  |
| Slowing | I feel I react too slowly to things that are said to me |  |  |  |  |
| Fatigue | I cannot keep an activity going for long |  |  |  |  |

Section: H


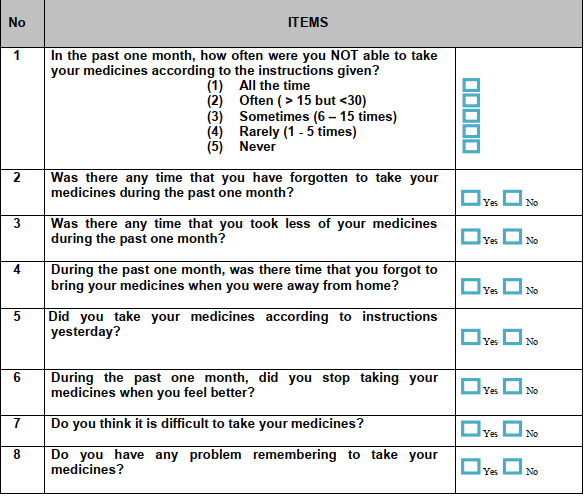


| Section: I  By putting a tick (☑) in one box in each group below, please indicate which statements best describe your own health state today. | |
| --- | --- |
|  |  |
| **Mobility** |  |
| I have no problems in walking around | ❑ |
| I have some problems in walking around | ❑ |
| I am confined to bed | ❑ |
|  |  |
| **Self-Care** |  |
| I have no problems with self-care | ❑ |
| I have some problems taking a bath/shower, or dressing myself | ❑ |
| I am unable to take a bath/shower, or dress myself | ❑ |
|  |  |
| **Usual Activities** (*e.g. work, study, housework, family or leisure activities)* |  |
| I have no problems with performing my usual activities | ❑ |
| I have some problems with performing my usual activities | ❑ |
| I am unable to perform my usual activities | ❑ |
|  |  |
| Pain / Discomfort |  |
| I have no pain or discomfort | ❑ |
| I have moderate pain or discomfort | ❑ |
| I have extreme pain or discomfort | ❑ |
|  |  |
| Anxiety / Depression |  |
| I am not anxious or depressed | ❑ |
| I am moderately anxious or depressed | ❑ |
| I am extremely anxious or depressed | ❑ |

|  |
| --- |
|  |
|  |
|  |
| To help people say how good or bad a health state is, we have drawn a scale (rather like a thermometer) on which the best state you can imagine is marked 100 and the worst state you can imagine is marked 0. |
|  |
| We would like you to indicate on this scale how good or bad your own health is today, in your opinion. Please do this by drawing a line from the box below (where ‘Your own health state today’ is written) to whichever point on the scale indicates how good or bad your health state is today. |

Best imaginable health state

9 0

8 0

7 0

6 0

5 0

4 0

3 0

2 0

1 0

100

0

Your own health state today

Worst imaginable health state
